# The Hasford Score May Predict Molecular Response in Chronic Myeloid Leukemia Patients: A Single Institution Experience 

**DOI:** 10.1155/2016/7531472

**Published:** 2016-10-12

**Authors:** Jarosław Dybko, Bożena Jaźwiec, Olga Haus, Donata Urbaniak-Kujda, Katarzyna Kapelko-Słowik, Tomasz Wróbel, Tomasz Lonc, Mateusz Sawicki, Ewa Mędraś, Agnieszka Kaczmar-Dybko, Kazimierz Kuliczkowski

**Affiliations:** ^1^Department of Hematology and Bone Marrow Transplantation, Wroclaw Medical University, Wroclaw, Poland; ^2^Department of Clinical Genetics, Collegium Medicum of Nicolaus Copernicus University, Bydgoszcz, Poland; ^3^Jagiellonian University Hospital, Department of Anaesthesiology and Intensive Care, Krakow, Poland; ^4^Lower Silesian Oncology Center, Department of Radiotherapy, Wroclaw, Poland

## Abstract

The Sokal, Hasford, and EUTOS scores were established in different treatment eras of chronic myeloid leukemia (CML). None of them was reported to predict molecular response. In this single center study we tried to reevaluate the usefulness of three main scores in TKI era. The study group included 88 CML patients in first chronic phase treated initially with standard imatinib dose. All of them achieved major molecular response (MMR) in time points defined by European LeukemiaNet (ELN). 42 patients lost MMR in a median time of 47 months and we found a significant difference in MMR maintenance between intermediate-risk (IR) and low-risk (LR) patients assessed by Hasford score. All 42 patients were switched to second-generation TKI (2G-TKI) treatment. At 18 months of 2G-TKI therapy we have still found a significant difference in BCR-ABL transcript levels and MMR rate between IR and LR groups. We did not find any of the described differences discriminating patients by Sokal or EUTOS score. In this retrospective single center analysis we found Hasford score to be useful in predicting molecular response in first chronic phase of CML patients.

## 1. Introduction 

Chronic myeloid leukemia (CML) has been a model disease for a variety of studies concerning scoring systems, graft versus leukemia effect, or tyrosine kinase inhibitors (TKI) treatment for many years. Scoring systems playing an important role in modern medicine to establish risk-adjusted optimal therapy [1] have been always essential for CML changing treatment modalities [1–3]. The three principal risk scores Sokal [2], Hasford [1], and European Treatment and Outcome Study (EUTOS) [3] were established in different eras of CML therapy with implications for prognosis and disease outcome [4]. Sokal and Hasford formula discriminated patients between high-risk, intermediate-risk, and low-risk groups but EUTOS score only between high-risk and low-risk groups. Sokal score was the first risk score metric designed for Ph+ CML. It was developed in chemotherapy era although still employed in quite recent trials like ENESTnd [5] or BELA [6] due to its proven usefulness for predicting survival in patients treated with imatinib [7] and second- generation TKI [8]. However Sokal score was not the perfect tool to properly discern low-risk and intermediate-risk patients survival during the first 3.5 years [1]. Hasford metric was designed based on data of patients treated with interferon alpha [1]. It was reported to predict the probability of 10-year overall survival in three risk group patients [9]. Originally the EUTOS score was successful to predict probability of complete cytogenetic response (CCyR) within 18 months of imatinib initiation and progression-free survival (PFS) for patients receiving imatinib [3]. In detail, Sokal and Hasford scores failed to differentiate CCyR rates between low-risk and intermediate-risk patients and the discrimination was significant only for CCyR rates at 18 months for high-risk patients [3] although both were successfully used to differentiate all risk patients treated with imatinib according to 5-year overall survival [10]. However, the usefulness of the EUTOS score in predicting survival and outcome in patients with early chronic phase CML treated with TKI was questioned [10, 11], although in other studies EUTOS score was reported to be potent in identifying patients with poor prognosis treated with imatinib (first or second line) or predicting long-term outcome [12–15]. In the TKI era none of available scores is reported to be useful in predicting molecular response. We were interested if any of them could be still employed. In our study we tried to find a correlation between Sokal, Hasford, and EUTOS score at the diagnosis and molecular response after TKI treatment of our patients. And surprisingly one of them worked.

## 2. Materials and Methods

### 2.1. Definitions

All patients were diagnosed in their first chronic phase and all of them achieved complete cytogenetic response (CCyR) at 12 months of imatinib treatment. Patients with advanced phases were originally excluded from the study. The Sokal score was calculated using the original formula: exp 0.016 × (age in years − 43.4) + 0.0345 × (spleen size in cm − 7.51) + 0.188 × ((platelet count/700)^2^ − 0.563) + 0.0887 × (blast cell percentage − 2.10) [2]. The Hasford score was calculated using the original formula: 0.6666 × age (0 when <50 years, 1 otherwise) + 0.042 × spleen size in cm + 0.054 × blast cell percentage + 0.0413 × eosinophil cells percentage + 0,2039 × basophil cells percentage (0 when <3%, 1 otherwise) + 1.0956 × platelet count (0 when <1500 × 10^9^/L, 1 otherwise) × 100 [1]. The Sokal risk score was designated as follows: low risk (score < 0.8), intermediate risk (score 0.8–1.2), and high risk (score > 1.2). The Hasford risk score was designated as follows: low risk (score ≤ 780), intermediate risk (score 781–1480), and high risk (score > 1480). The EUTOS score was also calculated using original formula: spleen size in cm × 4 + basophil cells percentage with low-risk (score ≤ 87) and high-risk (score > 87) groups designated [3]. Definitions of responses as well as time points evaluation and treatment were planned strictly according to European LeukemiaNet (ELN) recommendations including CCyR as no Ph+ cells in at least 20 metaphases analyzed in conventional cytogenetics of bone marrow aspirate, MMR as BCR-ABL^IS^ ≤ 0.10%, and MR^4.0^ as BCR-ABL^IS^ < 0.01% [16–18]. The study was approved by the Institutional Review Board and the local Ethics Committee.

### 2.2. Methods

#### 2.2.1. Cytogenetic Analysis

CC methods were performed at diagnosis on BM cells according to the standard protocols. Chromosome preparations were obtained from 24 h unstimulated (reference culture) and 48 h stimulated with granulocyte-macrophage colony-stimulating factor BM cell cultures in RPMI 1640. At least 25–30 G-banding with trypsin–Giemsa (GTG)-banded metaphases was analyzed for each patient. The karyotypes were described according to The International System for Human Cytogenetic Nomenclature (ISCN).

#### 2.2.2. Real-Time Quantitative Reverse-Transcriptase Polymerase Chain Reaction (RQ-PCR)

BCR-ABL expression was quantitated using real-time quantitative reverse-transcriptase polymerase chain reaction (RQ-PCR) according to Europe Against Cancer Protocol [19] using ABL as control gene. BCR-ABL/ABL ratio was expressed in percent and corrected to international scale (IS) by multiplying by correction factor established during external standardization.

## 3. Results

**Figure 1:**
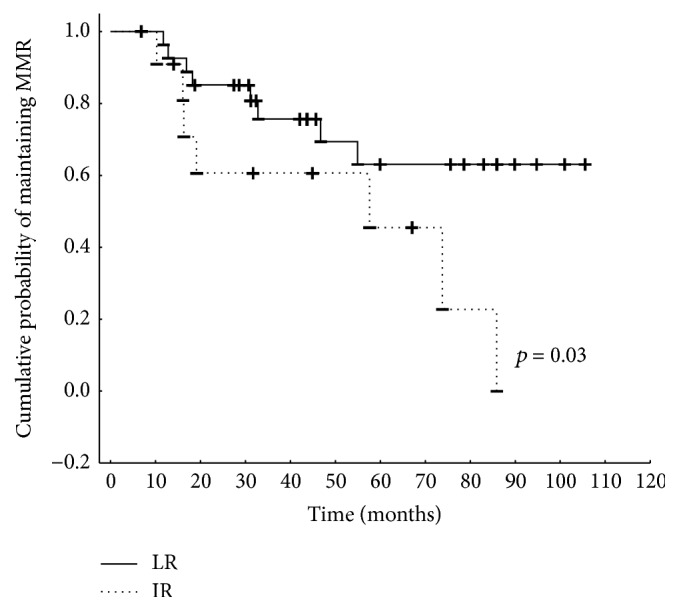
Cumulative probability of maintaining MMR on imatinib assessed by Hasford risk score (LR, low-risk group, IR, intermediate-risk group).

**Figure 2:**
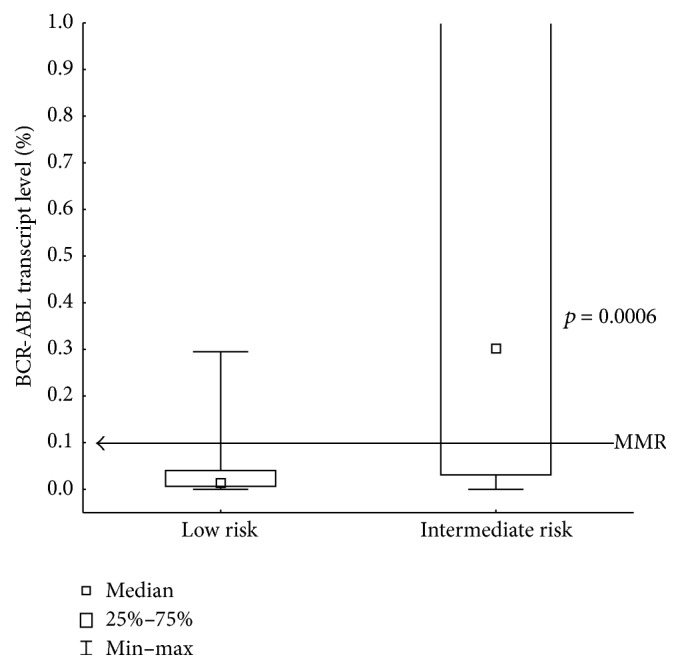
BCR-ABL transcript level after 3 months of 2G-TKI treatment assessed by Hasford risk score.

We analyzed a cohort of 88 patients (F/M: 42/46, median age 51 (21–83)) receiving standard dose imatinib treatment for first chronic phase of CML (Table 1). As assessed by Hasford risk analysis, the group comprised 57 low-risk (LR) and 31 intermediate-risk (IR) patients. In the initial group of patients, there were 5 high-risk patients who were excluded from the study. No additional chromosomal abnormalities were identified at baseline or any other time points. All patients achieved complete cytogenetic response (CCyR) and major molecular response (MMR) at time points defined by ELN. Of these, 42 patients lost MMR in a median time of 47 months but no BCR-ABL kinase domain mutations were detected. Within this group we identified 20 low-risk and 22 intermediate-risk patients. There was a significant difference in maintenance of the MMR between IR and LR patients (*p* = 0.03, Figure 1). This analysis revealed that all intermediate-risk patients lost MMR after approximately 85 months of imatinib treatment, while 62% of the low-risk patients maintained MMR throughout this time frame. During analysis, all 42 patients were switched to second-generation TKI (2G-TKI), dasatinib [20], or nilotinib [20] (Table 1). After 3 months of 2G-TKI treatment 19 patients of LR group (86%) and 9 patients of IR group (41%) achieved MMR. The median BCR-ABL transcript levels in the LR group were 0.01 (0.000–0.295) but in the IR group BCR-ABL levels were 0.301 (0.000–44.5) (*p* = 0.0006, Figure 2).

**Figure 3:**
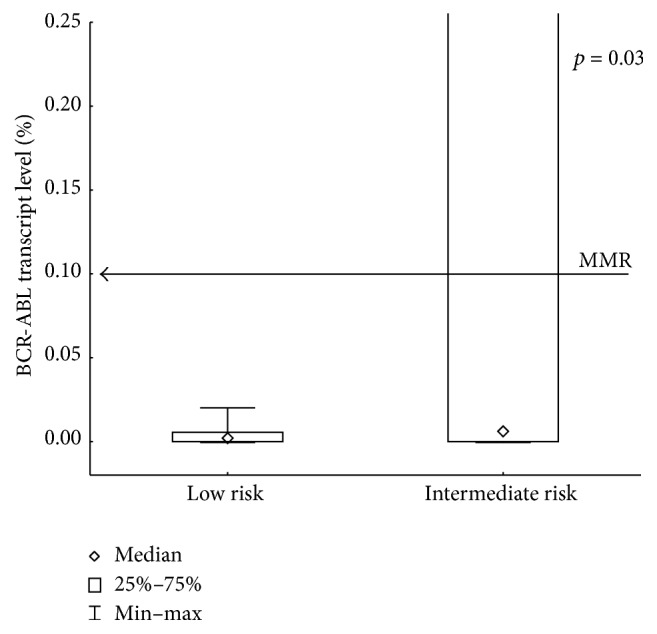
BCR-ABL transcript level after 18 months of 2G-TKI treatment assessed by Hasford risk score.

**Figure 4:**
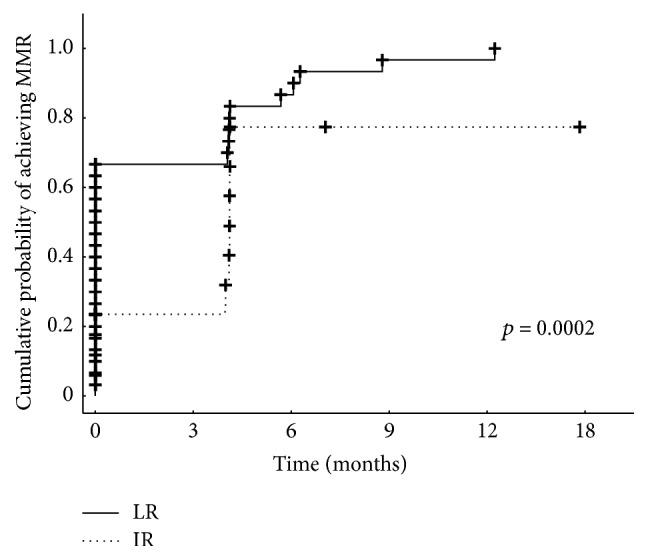
Cumulative probability of achieving MMR on 2G-TKI treatment assessed by Hasford risk score (LR, low-risk group, IR, intermediate-risk group).

**Figure 5:**
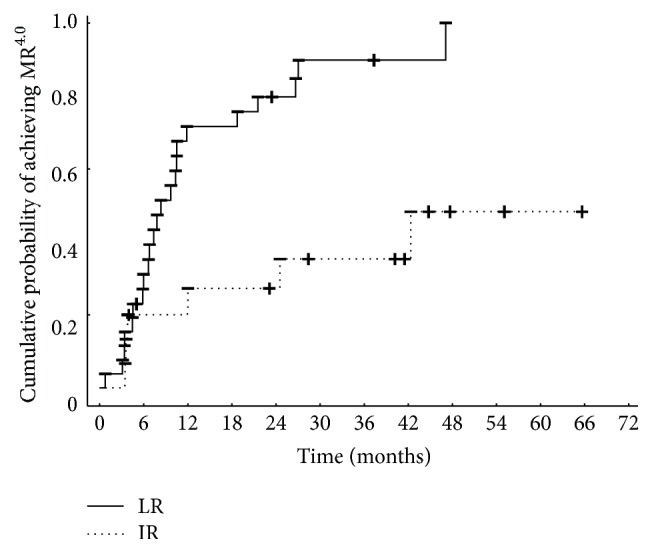
Cumulative probability of achieving MR^4.0^ on 2G-TKI treatment assessed by Hasford risk score (LR, low-risk group, IR, intermediate-risk group).

After 18 months of 2G-TKI treatment median BCR-ABL transcript levels in the LR group were 0.002 (0.00–0.02) but in the IR group BCR-ABL levels were 0.03 (0.000–21.1) (*p* = 0.03, Figure 3). All 20 low-risk patients achieved major molecular response (MMR). In the intermediate-risk group the response rate (MMR) was approximately 73% (16/22) and there is a significant difference in a probability of achieving MMR between groups (*p* = 0.0002, Figure 4). Longer follow-up revealed deep molecular response (MR^4.0^) differences between the groups. After 18 months of 2G-TKI treatment MR^4.0^ rate in LR and IR groups was 85.0% (17/20) and 36.3% (8/22), respectively. The probability of MR^4.0^ after 48 months of 2G-TKI treatment in LR and IR group was 100% and 51.7%, respectively (*p* = 0.01, Figure 5). We did not find any of the described significant differences discriminating patients by Sokal or EUTOS score (data not shown).

## 4. Discussion 

Our results are not so different from large studies results if we look closer at a long-term observation. In DASISION study molecular responses were estimated by Hasford score. Cumulative MMR incidence in dasatinib arm by 36 months in low-risk and intermediate-risk group was 83% and 65%, respectively [21]. In our study MMR incidence by 18 months in LR and IR group was 100% and 73%. Our results seem to be better but they are not directly comparable. Our 2G-TKI group was half-dasatinib and half-nilotinib and all patients were imatinib pretreated. In ENESTnd trial the highest rate of MR^4.0^ after 24 months of nilotinib treatment was observed in the group of patients (no prior imatinib exposure) with the lowest BCR-ABL transcript level after 3 months of this therapy (for subpopulations with 0.01 ≤ BCR-ABL^IS^ ≤ 1% and 1% ≤ BCR-ABL^IS^ ≤ 10%—MR^4.0^ rate after 24 months of treatment was 65.0% and 24.1%, resp.) [20]. In our study we observed similar responses after 24 months of 2G-TKI treatment. In LR group (median BCR-ABL^IS^ after 3 months of treatment—0.01%) and IR group (median BCR-ABL^IS^ after 3 months of treatment—0.301%) MR^4.0^ rate after 18 months of therapy was 85.0% and 36.3.1%, respectively. IR and LR groups may be equivalents of BCR-ABL^IS^ ranges in ENESTnd study as for MR^4.0^ achievements. It could indirectly confirm Hasford risk score and molecular response correlation in our observations. It would be interesting to compare the results from CA180-034 study describing long-term outcome with dasatinib after imatinib failure in chronic phase CML but the end points are progression-free survival (PFS) and overall survival (OS) rates only [22]. As mentioned before Hasford score was created to discriminate OS of CML patients treated with interferon alpha in three risk groups [1]. EUTOS score was able to assign high and low-risk groups of CML patients according to CCyR after 18 months of imatinib treatment [3]. Neither Hasford nor EUTOS score (derived using patients treated solely with imatinib) was able to predict molecular response and they were not intended to. There were not significant differences in achieving CCyR by 18 months between Hasford low and intermediate-risk groups patients treated with interferon alpha in large studies [9]. But they proved significantly higher probability of 10-year overall survival of low risk comparing to intermediate-risk patients [9]. We are aware of Hasford score limited usefulness in predicting MMR. As the Hasford metric was designed for assessing patients treated with interferon alpha, we found our results to be interesting and to be relevant to the discussion on optimizing scoring systems in chronic myeloid leukemia patients. If the observed difference between low and intermediate-risk patients in maintaining MMR on imatinib is confirmed, IR patients will become candidates for different first line treatment. Despite clinical studies, the choice between imatinib and second-generation TKI as the first line treatment remains an issue. Our results (if confirmed) promise to directly impact treatment decisions affecting IR patients.

## Figures and Tables

**Table 1:** Patients characteristics.

Characteristics	Value
Sex, no (%)	
Male	46 (52)
Female	42 (48)
Median age at diagnosis (range)	51 (21–83)
Hasford score at diagnosis, no (%)	
Low risk (LR)	57 (65)
Intermediate risk (IR)	31 (35)
Sokal score at diagnosis, no (%)	
Low risk	60 (68)
Intermediate risk	20 (23)
High risk	8 (9)
EUTOS score at diagnosis, no (%)	
Low risk	65 (74)
High risk	23 (26)
Hasford score, median value in LR group [min–max]	555 [0–766]
Hasford score, median value in IR group [min–max]	998 [415–1450]
MMR lost on imatinib (assessed by Hasford score) no (%)	
Low risk	20 (35)
Intermediate risk	22 (71)
Median time to MMR loss (range) [months]	47 (12–108)
2G-TKI, no (%)	
Dasatinib 100 mg	21 (50)
Nilotinib 800 mg	21 (50)
MMR at 3 months of 2G-TKI, no (%)	
Low risk	19/20 (86)
Intermediate risk	9/22 (41)
MMR at 18 months of 2G-TKI, no (%)	
Low risk	20/20 (100)
Intermediate risk	16/22 (73)
MR ≥ 4.0 at 3 months of 2G-TKI	
Low risk	10/20 (50)
Intermediate risk	4/22 (18)
MR ≥ 4.0 at 18 months of 2G-TKI	
Low risk	17/20 (77)
Intermediate risk	8/22 (37)

## References

[1] Hasford J., Pfirrmann M., Hehlmann R. (1998). A new prognostic score for survival of patients with chronic myeloid leukemia treated with interferon alfa. Writing Committee for the Collaborative CML Prognostic Factors Project Group. *Journal of the National Cancer Institute*.

[2] Sokal J. E., Cox E. B., Baccarani M. (1984). Prognostic discrimination in ‘good-risk’ chronic granulocytic leukemia. *Blood*.

[3] Hasford J., Baccarani M., Hoffmann V. (2011). Predicting complete cytogenetic response and subsequent progression-free survival in 2060 patients with CML on imatinib treatment: the EUTOS score. *Blood*.

[4] Hu B., Savani B. N. (2014). Impact of risk score calculations in choosing front-line tyrosine kinase inhibitors for patients with newly diagnosed chronic myeloid leukemia in the chronic phase. *European Journal of Haematology*.

[5] Saglio G., Kim D.-W., Issaragrisil S. (2010). Nilotinib versus imatinib for newly diagnosed chronic myeloid leukemia. *The New England Journal of Medicine*.

[6] Cortes J. E., Kim D.-W., Kantarjian H. M. (2012). Bosutinib versus imatinib in newly diagnosed chronic-phase chronic myeloid leukemia: results from the BELA trial. *Journal of Clinical Oncology*.

[7] Druker B. J., Guilhot F., O'Brien S. G. (2006). Five-year follow-up of patients receiving imatinib for chronic myeloid leukemia. *The New England Journal of Medicine*.

[8] Milojkovic D., Nicholson E., Apperley J. F. (2010). Early prediction of success or failure of treatment with second-generation tyrosine kinase inhibitors in patients with chronic myeloid leukemia. *Haematologica*.

[9] Hasford J., Pfirrmann M., Shepherd P. (2005). The impact of the combination of baseline risk group and cytogenetic response on the survival of patients with chronic myeloid leukemia treated with interferon alpha. *Haematologica*.

[10] Yamamoto E., Fujisawa S., Hagihara M. (2014). European Treatment and Outcome Study score does not predict imatinib treatment response and outcome in chronic myeloid leukemia patients. *Cancer Science*.

[11] Jabbour E., Cortes J., Nazha A. (2012). EUTOS score is not predictive for survival and outcome in patients with early chronic phase chronic myeloid leukemia treated with tyrosine kinase inhibitors: a single institution experience. *Blood*.

[12] Breccia M., Finsinger P., Loglisci G. (2012). The EUTOS score identifies chronic myeloid leukeamia patients with poor prognosis treated with imatinib first or second line. *Leukemia Research*.

[13] Tiribelli M., Bonifacio M., Calistri E. (2013). EUTOS score predicts long-term outcome but not optimal response to imatinib in patients with chronic myeloid leukaemia. *Leukemia Research*.

[14] Tao Z., Liu B., Zhao Y. (2014). EUTOS score predicts survival and cytogenetic response in patients with chronic phase chronic myeloid leukemia treated with first-line imatinib. *Leukemia Research*.

[15] Hoffmann V. S., Baccarani M., Lindoerfer D. (2013). The EUTOS prognostic score: review and validation in 1288 patients with CML treated frontline with imatinib. *Leukemia*.

[16] Baccarani M., Saglio G., Goldman J. (2006). Evolving concepts in the management of chronic myeloid leukemia: recommendations from an expert panel on behalf of the European LeukemiaNet. *Blood*.

[17] Baccarani M., Cortes J., Pane F. (2009). Chronic myeloid leukemia: an update of concepts and management recommendations of European LeukemiaNet. *Journal of Clinical Oncology*.

[18] Baccarani M., Deininger M. W., Rosti G. (2013). European LeukemiaNet recommendations for the management of chronic myeloid leukemia: 2013. *Blood*.

[19] Gabert J., Beillard E., van der Velden V. H. J. (2003). Standardization and quality control studies of ‘real time’ quantitative reverse transcriptase polymerase chain reaction of fusion gene transcripts for residual disease detection in leukemia—a Europe Against Cancer Program. *Leukemia*.

[20] Hochhaus A., Rosti G., Cross N. C. P. (2016). Frontline nilotinib in patients with chronic myeloid leukemia in chronic phase: results from the European ENEST1st study. *Leukemia*.

[21] Jabbour E., Kantarjian H. M., Saglio G. (2014). Early response with dasatinib or imatinib in chronic myeloid leukemia: 3-year follow-up from a randomized phase 3 trial (DASISION). *Blood*.

[22] Shah N. P., Guilhot F., Cortes J. E. (2014). Long-term outcome with dasatinib after imatinib failure in chronic-phase chronic myeloid leukemia: follow-up of a phase 3 study. *Blood*.

